# New Perspectives on Old and New Therapies of Staphylococcal Skin Infections: The Role of Biofilm Targeting in Wound Healing

**DOI:** 10.3390/antibiotics10111377

**Published:** 2021-11-10

**Authors:** Oriana Simonetti, Giulio Rizzetto, Giulia Radi, Elisa Molinelli, Oscar Cirioni, Andrea Giacometti, Annamaria Offidani

**Affiliations:** 1 Department of Clinical and Molecular Sciences Clinic of Dermatology, Polytechnic University of Marche, 60020 Ancona, Italy; grizzetto92@hotmail.com (G.R.); radigiu1@gmail.com (G.R.); molinelli.elisa@gmail.com (E.M.); a.offidani@ospedaliriuniti.marche.it (A.O.); 2 Department of Biomedical Sciences and Public Health Clinic of Infectious Diseases, Polytechnic University of Marche, 60020 Ancona, Italy; o.cirioni@staff.univpm.it (O.C.); a.giacometti@staff.univpm.it (A.G.)

**Keywords:** antimicrobial molecules, wound healing, staphylococcal skin infection

## Abstract

Among the most common complications of both chronic wound and surgical sites are staphylococcal skin infections, which slow down the wound healing process due to various virulence factors, including the ability to produce biofilms. Furthermore, staphylococcal skin infections are often caused by methicillin-resistant *Staphylococcus aureus* (MRSA) and become a therapeutic challenge. The aim of this narrative review is to collect the latest evidence on old and new anti-staphylococcal therapies, assessing their anti-biofilm properties and their effect on skin wound healing. We considered antibiotics, quorum sensing inhibitors, antimicrobial peptides, topical dressings, and antimicrobial photo-dynamic therapy. According to our review of the literature, targeting of biofilm is an important therapeutic choice in acute and chronic infected skin wounds both to overcome antibiotic resistance and to achieve better wound healing.

## 1. Introduction

Staphylococcal skin infections are one of the most common complications of both surgical sites [1] and chronic wounds, such as arterial, venous, and diabetic ulcers [2]. *Staphylococcus aureus* is one of the top four bacteria in terms of prevalence among chronic wounds [3,4,5], and its ability to produce biofilm is a major virulence factor contributing to wound chronicity and delayed healing [6]. Furthermore, *S. aureus* has a high public health impact due to increasing antibiotic resistance [7], and methicillin-resistant *Staphylococcus aureus* (MRSA) represents a therapeutic challenge, with an important role in slowing down the wound healing process [8,9,10,11].

A meta-analysis [6] of several studies reported that bacterial biofilm is present in 78.2% of chronic wounds and contributes to persistent infection. All staphylococcal species are responsible for 65% of persistent infections in chronic wounds [2] and can adhere to wound surface proteins to form colonies embedded in the biofilm that are resistant to antibiotic therapy [12].

Specifically, biofilm consists of microbial populations attached to a surface and immersed in a polymeric, hydrated extracellular fluid, known as extracellular polymeric substance (EPS) [13], which includes extracellular DNA (eDNA), polysaccharides, and proteins [14]. Furthermore, biofilm confers antiphagocytic capabilities, prevents the action of leukocytes [15], and can capture and make both complement and antibiotics ineffective, triggering persistent tissue damage and chronic inflammation [16]. Staphylococcal biofilm development follows a complex pathway that includes attachment, maturation, and dispersion, with an important role of environmental factors, such as the surface of adhesion considered and nutrients available [14].

The pathogenetic role of staphylococcal biofilm in wound healing was evaluated in a study by Roy et al. [17], showing that, in a porcine skin wound model, biofilm produced by *S. aureus* promotes degradation of collagen type 1 by repressing wound-edge miR-143 (a miRNA sensitive to staphylococcal biofilm) and consequently upregulating metalloproteinase-2 production. The collagen1/collagen3 ratio decreases, altering wound repair capacity, granulation tissue production, and promoting recurrence. This results in poorer healing outcomes and increased costs of care [18]. In addition, increased levels of metalloproteinases are typical in chronic wounds, which fail to heal effectively because the tensile strength of the type 1 collagen scaffold is lost [19]. Consequently, biofilm loading structurally compromises the wound tissue and promotes local infectious recurrence [19].

In addition to *S. aureus*, other types of Coagulase-negative staphylococci may also produce biofilms and be responsible for chronic wound maintenance, such as S. epidermidis [20] and *S. lugdunensis* [21]. However, there are different strains of staphylococci with varying degrees of biofilm production capacity, often in conjunction with other pathogens [22,23].

Biofilm frequently develops on the surface of wounds and is responsible for their chronicity [24]. Its removal can considerably improve the speed and quality of wound healing [25,26], using anti-biofilm agents. These are characterized by their low molecular weight and ability to break down biofilm by promoting penetration of antibacterial molecules and restoring leucocyte activity [27,28,29,30]. Given the high impact of chronic infected wounds [31,32] on public health and the increasing number of bacterial resistances to antibiotics, we reviewed the literature both to update the latest evidence on old antibiotics and molecules, and to highlight new molecules to overcome the staphylococcal defence strategies in order to improve wound healing.

We considered both recent and older molecules (Table 1), as we believe that all of them may be useful to overcome the growing antibiotic resistance and improve wound healing, particularly in association with antibiotics.

## 2. Results

### 2.1. Antibiotics

The gold standard for treatment of staphylococcal skin infections, including biofilm-forming strains, are systemic antibiotics. Beta-lactams are the first line therapy for both coagulase-negative staphylococci (CNS) and methicillin-sensitive *S. aureus* (MSSA) [60]. However, infections sustained by MRSA, or methicillin-resistant CNS, require an antibiogram to select the appropriate antibiotic [61]. Adjuvant therapy to eradicate the infection and improve wound healing is recommended in all conditions where there is potential for biofilm formation [60]. Since most antibiotics have been developed for bacteria in planktonic form (free-swimming), complementary strategies are required to target the biofilm and allow the antibiotic to reach the pathogen [62].

Conversely, incorrect use of antibiotics can promote *S. aureus* biofilm development. Some studies showed that concentrations below the minimum inhibitory concentration (MIC) of cephalothin [63], oxacillin [64], cephalexin [65], vancomycin [63], and Linezolid [66] can stimulate biofilm formation up to four-fold. Furthermore, Kaplan et al. [67] showed that ampicillin, cloxacillin, methicillin and amoxicillin used sub-MIC in vitro favoured biofilm formation in MSSA and MRSA strains by increasing eDNA release [68]. A similar phenomenon has been shown for S. epidermidis with tigecycline, novobiocin, linezolid, vancomycin and fluoroquinolones [69]. Finally, considering MRSA, Majidpour et al. [70] highlighted that vancomycin and azithromycin may have biofilm-inducing effects, while linezolid, clarithromycin, and cefazolin, followed by minocycline and clindamycin may be effective in inhibiting biofilm formation.

#### 2.1.1. Beta-Lactams

The activity of beta-lactams in wounds infected with biofilm-producing staphylococci may be compromised, requiring combination with other molecules or procedures [33]. In addition, low-dose beta lactam can promote S. aureus biofilm production, which is dependent on the level of eDNA. The latter plays the role of matrix adhesin in the biofilm [71,72,73,74,75] and is produced by bacterial cell lysis determined by the autolysin AtlA [73]. Sub-MIC beta lactams appear to induce AtlA and thus increase biofilm production [67].

In an in vitro study by Kirmusaoglu et al. [33], resistance to beta lactams in biofilm-producing strains of MRSA and MSSA is overcome through synergistic action with 2-aminothiazole, highlighting a possible solution to the treatment of biofilm-forming staphylococcal infections.

#### 2.1.2. Macrolides

Macrolides are bacterial protein synthesis inhibiting molecules with an unclear role against staphylococcal biofilm [76]. We found some studies suggesting their antibiofilm activity only when in combination with other antibiotics, as clarithromycin/daptomycin, [76] clarithromycin/vancomycin [77], and roxithromycin/imipenem [78]. In vivo studies referring specifically to skin wound healing and biofilm are lacking.

#### 2.1.3. Teicoplanin

Teicoplanin is a glycopeptide and represents the antibiotic of choice in empirical therapy of MRSA infections [79]. As it is an effective molecule in chronic wounds; it is often the comparator in animal models and shows excellent results in both preventing biofilm formation and treating wounds with an established biofilm. It also demonstrates to be effective in promoting the wound healing process [34].

#### 2.1.4. Daptomycin

Daptomycin is a lipopeptide with activity against Gram-positive bacteria, including MRSA and vancomycin-resistant *S. aureus* [80,81,82,83], and is recommended for the treatment of skin, soft tissue, and bloodstream infections. It also has a strong activity against staphylococcal biofilm [84].

In a study [35] in a mouse model with MRSA-infected burns, the efficacy of intraperitoneal daptomycin was evaluated in comparison to intraperitoneal teicoplanin and a no-treatment control. The best antimicrobial activity and histological outcome were obtained in the daptomycin-treated group.

#### 2.1.5. Tigecycline

Tigecycline is a glycylcycline antibiotic that has demonstrated efficacy against staphylococci, particularly MRSA, and their biofilms, although the best results have been obtained in combination with other antibiotics [85,86,87,88,89]. However, a direct effect of tigecycline on wound healing of *S. aureus*-infected wounds in mice was shown through modulation of matrix metalloproteinase-9 expression, proving superior to teicoplanin in comparison [36].

#### 2.1.6. Dalbavancin

Dalbavancin is a novel lipoglycopeptide with a spectrum of action against Gram-positives and shows great penetration of staphylococcal biofilm [90,91,92].

Its role in wound healing was investigated in a mouse model with MRSA-infected wounds [38]. The comparison was made with daily vancomycin (10 mg/kg) and dalbavancin with two administrations on day 1 and day 8. At 14 days, both antibiotics had reduced the bacterial load, with dalbavancin being more effective, which also resulted in healing with normal, well-organised keratinized epithelium, slightly less than the uninfected group but better than the vancomycin-treated group. In addition, epidermal growth factor receptor (EGFR) and vascular endothelium growth factor (VEGF) values were found to be higher than with vancomycin.

### 2.2. Quorum Sensing Inhibitors

Quorum sensing (QS) is a cell-to-cell bacterial communication mechanism capable of regulating gene expression according to environmental conditions [93]. *Staphylococci*, particularly *S. aureus*, are able to regulate their virulence factors, including biofilm and toxin formation, thanks to QS [94,95,96,97,98,99,100,101,102]. By using molecules that can inhibit QS it is possible to circumvent the adaptation of staphylococcal strains to the wound conditions, preventing the formation of biofilm [103,104,105,106,107].

#### 2.2.1. RIP

RNA III inhibiting peptide (RIP) is a seven-amino acid molecule capable of inhibiting the synthesis of RNAIII, a transcriptional unit of the staphylococcal accessory gene regulator (Agr) system responsible for QS and biofilm formation [108,109].

This peptide has been proven to be effective in treating device-associated infections due to MRSA and MSSA [110,111,112,113], and its role in skin wound healing in animal models has also been evaluated. Schiele et al. [39] showed in a mouse model that *S. aureus* and *S. epidermidis* biofilm reduced the rate of wound healing significantly compared with an uninfected wound, with a histologically lower degree of re-epithelisation. Infected mice treated with topical RIP 100 mcg for 7 days had a histologically comparable degree of healing to uninfected mice. This underlines the possible topical role of RIP in preventing biofilm and ensuring better wound healing.

The role of topical RIP (20 mcg) in the healing of MRSA-infected wounds in combination with daily teicoplanin (7 mg/kg) for 7 days compared with teicoplanin alone has also been demonstrated in a mouse model, resulting in a lower bacterial load and histological degree of healing (collagen, re-epithelisation, microvascular density and expression of VEGF comparable to non-infected wounds [40]. It is also hypothesised that RIP may induce VEGF by improving the quality and speed of wound healing [39,40,112,113,114].

Topical daily RIP was also used in two cases [115] of patients with chronic diabetic ulcers after failure of conventional antibiotic therapy, in systemic combination with systemic daptomycin, avoiding amputation of the lower limb.

#### 2.2.2. F19, F12 and F1

F19, F12 and F1 are small-molecule biaryl hydroxyketones that inhibit staphylococcal QS and thus also affect biofilm production [116]. Their efficacy was evaluated in a study by Kuo et al. [41] in an animal model. F19, F12 and F1 were applied by injection (20 mg/kg) into MRSA-infected insect larvae, while F12 and F1 were applied topically to mice with MRSA-infected wounds. F19, F12 and F1 provided a survival advantage for treated infected larvae over untreated larvae. In the mouse model, F12 and F1 improved the speed of wound healing. In addition, it was shown that some antibiotics ineffective in monotherapy against MRSA, such as cephalothin and nafcillin, can be restored in combination with F12 and F1, observing a MIC reduction in vitro by 40 and 60 times respectively.

#### 2.2.3. FS10

FS10 is a tetrapeptide with the same mechanism of action as RIP and with enhanced antistaphylococcal activity against MRSA [37,117,118]. Its role in wound healing was assessed histologically in a mouse model, showing that the combination of systemic tigecycline (7 mg/kg) and topical FS10 (20 µg) resulted in better healing and control of both MRSA and MSSA infection than monotherapy [119]. FS10 shows the best results in combination with a systemic antibiotic, particularly tigecycline. The latter can accelerate wound healing by reducing expression of matrix metalloproteinase 9 but is inferior to combination therapy [42]. FS10 and QS inhibitors in general act by inhibiting staphylococcal virulence factors, including biofilm formation.

### 2.3. Antimicrobial Peptides

Antimicrobial peptides (AMPs) are a large group of molecules that are generally part of the innate immunity of all life forms and are becoming increasingly important in the era of antibiotic resistance [120]. Their mechanism of action differs depending on the type of AMPs and may result in killing bacteria by lysis or by targeting intracellular components [121]. In addition, AMPs have also shown immunomodulatory properties, reducing the inflammatory component, inducing epithelial cell migration and neoangiogenesis [122,123,124,125]. Their role in local biofilm inhibition and wound healing has been shown in the literature [126,127,128], allowing destruction of the polymeric matrix biofilm by binding with anionic bacterial Lipopolysaccharides [129].

#### 2.3.1. Innate Defence Regulator (IDR)-1018

IDR-1018, a synthetic cationic peptide, showed strong antibiofilm action by promoting degradation of the nucleotide guanosine penta- and tetra-phosphate (p)ppGpp, which regulates biofilm formation in staphylococci and participates in antibiotic resistance [130,131]. In a study on a mouse model with *S. aureus*-infected wounds, IDR-1018 injected subcutaneously was shown to reduce bacterial load and eliminate biofilm [43].

#### 2.3.2. LL-37

LL-37 is a natural cathelicidin-derived AMP that is important in both inhibiting staphylococcal biofilm formation and wound healing. LL-37 is present in non-infected wounds and promotes the reparative process [132], whereas the presence of antibodies to LL-37 inhibits re-epithelisation and its expression is reduced in chronic wounds [133]. Adenovirus-mediated gene transfer for LL-37 was observed to result in improved wound healing in obese mice [44]. In addition, LL-37 appears to promote non-infected wound healing in a dexamethasone-treated mouse model [45].

The anti-biofilm activity is due to the prevention of its formation by impairing bacterial adhesion and staphylococcal QS, while also increasing bacterial motility [46]. A limitation of this AMP is that it can be inactivated by endogenous, bacterial proteases and sub-physiological salt concentrations [46,47,48,49,134].

#### 2.3.3. SHAP1

SHAP1 is a synthetic peptide (APKAMKLLKKLLKLQKKGI) that has shown excellent antimicrobial activity against both bacteria and fungi, maintaining great stability both in the presence of proteases and in a salty environment [50]. One relevant feature is its ability to promote wound healing both in vitro and in the S. aureus-infected mouse model by activating the EGFR pathway. It proved superior in this compared to LL-37, which is inactivated in a salty or protease-rich environment [50,135,136]. SHAP1, applied topically (1µM) to the wounds of S. aureus-infected and uninfected mice, accelerated the healing process in both cases. Complete closure was achieved in 3 days [50].

#### 2.3.4. DRGN-1

DRGN-1 is a synthetic cationic AMP derived from an H1 histone of Komodo dragon and has been shown to be effective as an anti-biofilm, anti-staphylococcal and wound healing agent in a mouse model [51]. Topically applied DRGN-1 showed to promote keratinocyte migration in vitro and act on the EGFR-Signal transducer and activator of transcription (STAT)1/3 pathway, while its antimicrobial activity consists of permeabilising bacterial cell membranes and disrupting biofilm [51].

#### 2.3.5. Dermaseptin Peptide2 (DMS-PS2)

DMS-PS2 is a synthetic cationic AMP belonging to the dermaseptins with potent anti-biofilm, anti-MRSA and wound healing actions [52]. In a mouse model with MRSA-infected wounds, DMS-PS2 was applied topically, resulting in a drastic reduction in the bacterial load after 1 day and a completely increased rate of re-epithelialisation compared with untreated wounds.

#### 2.3.6. Cell-Free Supernatant (CFS) of *Lactobacillus plantarum* USM8613

In this study, a CFS of *Lactobacillus plantarum* USM8613 was proven to inhibit *S. aureus* growth and biofilm formation on a porcine skin wound model by increasing beta-defensin levels. The protein-rich fraction of *L. plantarum* USM8613 was shown to be effective in promoting wound healing in a mouse model, and it increased the immune response and cytokine and chemokine production [137].

#### 2.3.7. Def-1

Honey defensin-1 is a recombinant molecule derived from honey (honeydew and manuka type) and was tested in a chronic wound model with biofilm formed by various pathogens, including *S. aureus*. Def-1 was effective in significantly reducing mature biofilm by 24 and 48 h [53]. In vivo studies to confirm its action on wound healing are still not available.

### 2.4. Other Topical Dressing

#### Octenidine Dihydrochloride (OCT)

OCT is a cationic surfactant with antiseptic properties. OCT-impregnated gauze dressing was used in a murine model with MRSA-infected skin wounds, resulting in accelerated wound healing after 24 h compared to phosphate-buffered saline controls and the 2% mupirocin group. In addition, histologically fewer inflammatory cells, better re-epithelialisation, and more mature collagen fibres were found in the OCT group [54].

### 2.5. Antimicrobial Photo Dynamic Therapy (APDT)

APDT may represent an alternative for the treatment of localised skin infections, demonstrating in vitro efficacy for different types of micro-organisms [138]. Its mechanism of action is based on exposing the bacteria-infected surface to light of a defined wavelength, after applying a photosensitiser capable of inducing reactive oxygen species and leading to a cytotoxic effect against the pathogens [138,139,140,141,142,143,144,145,146,147,148].

#### 2.5.1. RLP068/Cl

RLP068/Cl is a photosensitiser derived from tetracationic Zn (II) phthalocyanine [149] that was used in a biofilm-producing MRSA-infected mouse model with 689 nm light. A single session of APDT was performed 2 days after infection, following biofilm production, with a marked reduction in bacterial load on the same day compared to untreated subjects and those treated with systemic teicoplanin (7 mg/kg). At 7 days after therapy, the reduction in bacterial counts was similar between APDT and teicoplanin, suggesting the efficacy of RLP068/Cl in controlling infection. In addition, histological evaluation showed complete re-epithelialisation in APDT-treated mice at 7 days and good healing in the teicoplanin group. This highlighted the role of RLP068/Cl in treating chronic infections with significant biofilm formation [55].

#### 2.5.2. Curcumin Encapsulated in Silica Nanoparticles

Cucurmin is known to be a photosensitiser [150,151,152,153,154], and an encapsulated form of it in silica nanoparticles was used with APDT in an in vitro study on human fibroblast cultures, demonstrating its efficacy in reducing both the bacterial count of S. aureus and the production of biofilm, while also allowing improved human fibroblast activity [56].

#### 2.5.3. Aminolevulinic Acid (ALA)

A study by Lin et al. [57] showed the efficacy of ALA in three patients with chronic leg ulcers resistant to conventional therapy. One to three sessions of APDT were carried out, and after the first session no more staphylococci or other bacteria were isolated. The patients did not present recurrences for 29 months, highlighting the action on wound healing.

#### 2.5.4. Hypericin Nanoparticles

Hypericin is a natural photosensitiser with also an antibacterial and anti-biofilm action [155,156,157,158]. Nanoparticles are an effective vehicle that makes it possible to limit the lipophilicity and therefore the toxicity of this photosensitiser to the microbial target only [157,158,159,160]. In a study by Nafee et al. [58], the efficacy of hypericin nanoparticles on MRSA was evaluated in two models. In the in vitro model, an excellent ability to inhibit biofilm formation was demonstrated, and in the murine model, infected and treated wounds healed faster and histologically better than untreated wounds.

#### 2.5.5. Methylene Blue aPDT (MB-aPDT)

In a study by Pérez et al. [59], the efficacy of MB-aPDT alone or in combination with mupirocin was evaluated in a mouse model of a *S. aureus*-infected wound. A single session with MB-aPDT alone showed better cosmetic (no residual scarring) and healing (loss of crusts and reduction in extent) results than untreated control. Furthermore, MB-aPDT determined histological evidence of improved connective tissue production and cellularity in the dermis compared to mupirocin alone. The combination of mupirocin and MB-aPDT did not improve the effect on wound healing.

## 3. Discussion

In our review of the literature, we evaluated the efficacy of targeting staphylococcal biofilms in skin wounds, considering mainly the effect on wound healing in in vivo studies, where histological assessment was also reported. For this reason, studies without skin histological evaluation were not included, while others with in vitro relevance (e.g., human fibroblast) were cited. Therefore, considering exclusively the selection criteria, this review is not exhaustive for every existing therapy that could have an anti-biofilm effect and represents a selection of all available studies.

Regarding antibiotics, beta lactams are indicated in combination with adjuvant therapy for chronic wounds with biofilm-forming staphylococcal infection [33]. To the best of our knowledge, in vivo studies with histological assessment of wound healing in wounds infected with biofilm-producing staphylococci are lacking. However, many adjuvants showed histological benefits in wound healing, and thus, potentially, all the topical therapies described previously may be suitable. (Table 1) Further studies are needed to provide histological evidence of the efficacy of these combinations in wound healing. In addition, teicoplanin, daptomycin and dalbavancin also showed to improve wound healing in vivo in mouse models. For tigecycline better control of infection was achieved in combination with daptomycin or rifampicin, although superiority to teicoplanin in wound healing was demonstrated through modulation of metalloproteinase-9 in a mouse model infected with biofilm-producing *S. aureus* [36]. This may suggest that tigecycline has a greater effect on wound healing than one of the leading antibiotics for MRSA, teicoplanin, but both antibiotics are effective in either controlling infection or promoting better wound healing. In our opinion, tigecycline or teicoplanin may be used in biofilm-forming MRSA skin infections as first line. In the case of non-responsive and non-healing infections, we suggest evaluating the combination with one of the described adjuvant therapies. However, most of these have never been tested in humans and are not readily available.

For example, quorum sensing inhibitors are also promising treatment options, but there is a lack of studies in humans to assess their efficacy. RIP has been used topically in two cases of chronic diabetic ulcers in combination with daptomycin, with clinical improvement [115]. Although they have been shown to modulate VEGF expression and wound healing, the best results were obtained in combination with systemic antibiotics, such as FS10 and tigecycline. These molecules can help increase MRSA sensitivity towards antibiotics to which they are normally resistant. Specifically, F12 and F1 reduced the MIC for cephalothin and nafcillin by about 50-fold [41]. Increased use of these molecules topically, especially in association with a systemic antibiotic, may therefore both promote wound healing and increase the eradicating capacity of the antibiotic used.

Similarly, better results are possible for AMPs in combination with systemic antibiotics. In our review of the literature, many studies do not consider the histological evaluation of wound healing, preventing an assessment of their effect on the healing process and their direct correlation with the anti-biofilm action. However, many molecules have been referred with excellent potential against the staphylococcal biofilm and represent an important therapeutic possibility worthy of further studies, including applications on humans [43,44,45,46,47,48,49,50,51,52,53,54,55,56,57,58,59,132,133,134,135,136,137,138,139,140,141,142,143,144,145,146,147,148,149,150,151,152,153,154,155,156,157,158,159,160,161,162,163,164].

For instance, naja atra cathelicidin (NA-CATH):ATRA1-ATRA1, a highly cationic synthetic peptide derived from a natural snake cathelicidin, showed exceptional anti-biofilm properties against *S. aureus* [161,162] and has been found to be superior to LL-37 in inhibiting biofilm under saline environmental conditions [162]. This would overcome the limitations of using LL-37 due to environmental factors, but studies demonstrating an effect on wound healing are still lacking. We believe that this molecule deserves further in vivo studies before clinical application.

Better results on biofilm control can be achieved by combining AMPs with different mechanisms of action, as well as AMPs and antibiotics. In a mouse model study [165], topical RIP plus Temporin A, an AMP effective against biofilm and promoting wound healing [166], was shown to result in better control of glycopeptides-intermediate *S. aureus*-infected wounds compared with monotherapy and rifampicin. This study suggests that the combination of an anti-QS agent and a specific anti-biofilm agent further increases efficacy against antibiotic-resistant staphylococci. Another interesting molecule is 1.037, a synthetic peptide of 9 amino acids, which can inhibit biofilm formation in staphylococci by increasing bacterial motility and reducing the expression of genes responsible for biofilm formation and QS but has little antimicrobial activity [161]. Even in this study, data on an in vivo model of wound healing are lacking. In our opinion, the combination of AMPs and other molecules with a different mechanism of action could be, in the next future, the solution for difficult multifailure conditions.

We also want to mention bacteriocins, ribosomally synthesized bacterial peptides with important activity against staphylococcal biofilm. For example, Garvicin KS and microcin P1 showed in vitro anti-biofilm activity against both MSSA and MRSA. It was also observed that the combination of these two bacteriocins reduced MRSA resistance to penicillin G, revealing both antibiofilm and anti-MRSA activity [167]. The use of these molecules may therefore help to make antibiotics effective against resistant strains, although there is a lack of studies on wound healing.

With regard to *S. epidermidis*, one study [168] showed that Nisin A M17Q, a bacteriocin-derivative produced by Lactococcus lactis, can inhibit biofilm formation and the growth of *S. epidermidis* in an in vitro wound model more than wild type Nisin A. [168] This is one of the few studies considering the action of biofilm-producing *S. epidermidis* on wounds, although in an in vitro model [168]. Further animal studies and comparisons with other promising molecules are needed to assess its effective action on wound healing.

Other molecules have promising actions against staphylococci in the literature, in particular, defensins, proteins that mediate the innate immunity of organisms against bacteria. One example is the defensin-like peptide (DLP)-P2, a fungal-derived molecule that was shown to be effective in controlling multidrug-resistant *S. aureus* infections and biofilm formation both in vitro and in a mouse model with peritoneal infection [169]. A study of wound healing has not been carried out, but the prospects for using this molecule are interesting, especially in cases with extreme resistance to antibiotics.

Regarding the most common topical dressings already known, in a study by Brackman et al. [170] their anti-biofilm efficacy was evaluated in an in vitro model of *S. aureus* and *S. epidermidis*. Referring to inhibition of biofilm formation, dressings without antimicrobial agents and with only alginate fibres, carboxymethylcellulose (CMC), cotton, or hydrocellular foam proved ineffective or poorly effective. Conversely, dressings with antimicrobial agents such as povidone-iodine (PVP-iodine), hydroactive colloid gel, silver dihydrogen citrate, fusidic acid (20 mg/g) and polyhexanide have been effective in preventing biofilm formation. Finally, dressings containing ionic silver, metallic silver and silver sulphate, silver sulphadiazine, PVP-iodine (10 g/100 mL) and ozonated olive oil were effective in inhibiting biofilm formation.

With regard to biofilm-eradicating activity, fusidic acid, ozonated olive oil and silver dihydrogen citrate were effective for both *S. aureus* and *S. epidermidis*, while Betadine (0.1% *w*/*w*) with polyhexanide (0.1% *w*/*w*) and Hydroactive colloid gel were effective only for S. epidermidis. However, these dressings alone are often not sufficient to eradicate infection, and the possibility of combining an antibiotic or AMPs may improve the outcome of wound healing. Again, in vivo studies are required to evaluate efficacy. We suggest the early use of dressings with silver derivates, PVP-iodine or ozonate olive oil, considering their widespread availability.

An important role in the treatment of wounds infected with biofilm-producing staphylococcal strains is played by APDT, which was proven, in association with RLP068/CI, [152] to be faster than teicoplanin in reducing bacterial load and more effective in wound healing on human fibroblast cultures. In vivo studies are needed to evaluate its efficacy in wound healing. In the literature, we found a case report of APDT with ALA on a patient with a chronic ulcer infected with *S. aureus* resistant to conventional therapy, with excellent clinical results on wound healing and a long period without relapse (29 months) [158]. This suggests that in the most difficult cases, APDT may be a valid treatment option. There are promising studies in the literature demonstrating in vitro anti-biofilm staphylococcal action of APDT with various photosensitisers and blue light, [171,172,173,174,175,176,177,178,179] but which are lacking in vivo histological evaluation of wound healing.

Other promising treatments have not been investigated because there is no evaluation of wound healing, such as antibodies against staphylococcal antigens [174,175,176,177,178], nanotechnology [179,180,181,182], and new genetic approaches [183,184]. In addition, bacteriophages are viruses with a predatory action against specific bacteria [185]. Their effectiveness in eradicating biofilms has been evaluated in combination with other molecules, such as antibiotics [186,187,188]. It was also shown that the bacteriophage phiIPLA-RODI can be useful in eradicating *S. aureus* biofilms 24 h per day both in vitro and in an ex vivo pig model, especially in combination with a phage-derived lytic protein CHAPSH3b. The latter resulted in a reduction in the *S. aureus* population up to 7 h after exposure, followed by bacteriophage activity, which limits bacterial regrowth [185]. All of these methods have been shown to have an anti-biofilm effect, but no studies were conducted to assess their impact on wound healing.

Currently, only RIP and APDT with ALA were used in human patients, albeit only in case reports. In our opinion, these are promising and will help to overcome bacterial resistance, especially in difficult multifailure cases. We hope that other therapies will soon be proved in humans, allowing the treatment of chronic wounds with difficult tissue healing.

## 4. Materials and Methods

A narrative review of the literature was performed on Pubmed using as keywords individually or in combination: antistaphylococcal drugs, antistaphylococcal peptides, antistaphylococcal therapy, staphylococcal skin infection, staphylococcal, treatment, quorum sensing, biofilm, and wound healing. The aim of this review is to bring together the latest evidence on new and old anti-staphylococcal therapies, assessing their anti-biofilm properties and their effect on skin wound healing. We gave more consideration to studies performed in vivo with a histological evaluation of wound healing. Only English-language studies were included.

We did not consider a timeframe or a time limit, since even if a molecule was old, it would still be possible to use it to overcome growing bacterial resistance, maybe in combination with an antibiotic.

## 5. Conclusions

From our review of the literature, prevention and eradication of biofilm is an important therapeutic target in acute and chronic infected skin wounds both to achieve better wound healing and to overcome antibiotic resistance.

The best therapeutic and tissue-repair effect is obtained by combining systemic antibiotic therapy with a local agent that can act directly on the biofilm by breaking it down or preventing its formation. One of the limitations of the reviewed data is that most of them refer to in vitro studies or animal models, and human studies lack adequate sample size.

In conclusion, targeting biofilm can be an effective strategy not only to overcome antibiotic resistance conditions but also to significantly improve the outcome of wound healing.

## Figures and Tables

**Table 1:** Summary of reviewed therapies.

	Therapy	Experimental Model	Wound Healing Assessment	Histological Findings	Advantages/Disadvantages
ANTIBIOTICS					
Kirmusaoglu S. et al., 2020 [33]	Beta-lactams	In vitro, MRSA ATCC43300, MRSA, MSSA, beta-lactams combined with 2-aminothiazole as adjuvant	Not available	Not available	- First line treatment for MSSA and CNSA - Low efficacy alone versus biofilm-producing strains, needed association with adjuvants - Sub- MIC concentration may induce biofilm production [34]
Simonetti et al., 2008 [34]	Teicoplanin	In vivo murine model with MRSA infected skin wound; placebo vs. aPDT vs. aPDT + RLP068 vs. teicoplanin intra peritoneal (i.p.) vs. non infected	Better wound-healing response compared to placebo	- complete and normal epithelialization, - thick granulation tissue - regular collagen deposition	- First line treatment for MRSA - Improve wound healing - aPDT + RLP068 showed better results for wound healing
Simonetti et al., 2017 [35]	Daptomycin	In vivo murine model with *S. aureus* ATCC43300 infected skin wound (burn); daptomycin i.p. vs. teicoplanin i.p. vs. placebo vs. non infected	Better overall healing of Daptomycin group	- better epithelization - significantly higher collagen scores - higher immunohistochemical expression of wound healing markers (EGFR and FGF-2)	- better in vivo efficacy than teicoplanin - treatment option in more serious cases
Simonetti et al., 2011 [36]	Tigecycline	In vivo murine model with *S. aureus* ATCC43300 infected skin wound (burn); uninfected control group vs. infected no treatment vs. tigecycline i.p. vs. teicoplanin i.p.	Tigecycline showed better impact on wound healing	- Significant decrease in MMp-9 expression - Faster re-epithelisation vs. teicoplanin - Earlier Collagen better organised in the dermis vs. teicoplanin - Poor inflammatory response	- Modulatory effect on MMP-9 expression and accelerated wound healing compared to teicoplanin - Therapeutic option in more severe cases - Improved wound healing in combination with topical FS10 [37]
Simonetti et al., 2020 [38]	Dalbavancin	In vivo murine model with MRSA infected skin wound; vancomycin i.p. vs. dalbavancin i.p. vs. uninfected vs. untreated	faster healing after dalbavancin treatment	- robust epidermal coverage, regular and keratinized epidermal lining - well-organized granulation tissue with numerous blood vessels - Immunohistochemistry with higher levels of EGFR and VEGF, reduction of MMP-9 and MMP-1	- Faster wound healing than vancomycin - Treatment option in case of vancomycin resistance - Both effective in controlling infection
QUORUM SENSING INHIBITORS					
Schierle et al., 2009 [39]	RIP	In vivo murine model with *S. aureus* and S. epidermidis biofilm producers; uninfected vs. RIP topically (100 mcg for 7 days) vs. untreated	Better wound healing vs. untreated	- Re-epithelialization significantly more rapid with RIP	- Topical RIP restores normal wound healing kinetics - Useful only in localized infections
Simonetti et al., 2008 [40]		In vivo murine model with MRSA infected skin wound; topical RIP (20 mcg), teicoplanin i.p., allevyn, allevyn + teicoplanin i.p., topical RIP + teicoplanin i.p.	Better wound healing with topical RIP + teicoplanin	- only topical RIP + teicoplanin restored epithelial, granulation, and collagen scores, as well as microvessel density and VEGF expression	- Possible association with systemic antibiotic (teicoplanin) improves both infection control and wound healing - Alternative to overcome antibiotic resistance - RIP can induce VEGF, improving quality and speed of wound healing - Only 2 case reports on chronic diabetic ulcers in combination with daptomycin
Kuo et al., 2014 [41]	F19,F12, and F1	In vivo (1) MRSA-infected insect larvae; F19, F12 and F1 injection (20 mg/kg) (2) in vivo murine model with MRSA-infected wounds; topical F12 and F1 vs. untreated	(1) F19,F12, and F1 improved survival of larvae (2) F12 and F1 improved the speed of wound healing	- not available, only wound size considered	- Useful in improving wound healing - Possible contribution in reducing MICs by 50-fold for resistant antibiotics, such as cephalothin and nafcillin
Simonetti et al., 2016 [42]	FS10	in vivo murine model with MRSA and MSSA-infected wounds; topical FS10 (20mcg) + tigecycline i.p. (7 mg/kg) vs. monotherapy vs. untreated vs. uninfected	FS10 + tigecycline showed better wound healing and infection control	- robust epidermal coverage, regular epidermal lining, evident keratinization - the dermal papillae were still few. - Thick granulation tissue with many vessels and fibres - Collagen more organized and regular collagen fibres - Not evident inflammatory response	- Improvement even in FS10 monotherapy comparable to tigecycline monotherapy - Best result in combination therapy
ANTIMICROBIAL PEPTIDES					
Etayash H. et al., 2020 [43]	IDR-1018	In vivo murine model with MRSA infection abscess; IDR-1018 injected subcutaneously	Not available	Not available	- Reduction of bacterial load and elimination of biofilm - Further studies are needed to confirm action on wound healing
Carretero M. et al., 2008 [44]	LL-37	In vivo murine model non infected wound, adenoviral transfer of LL-37	Improved wound healing compared to untreated	- Significant increase in re-epithelialisation - Significant increase in granulation tissue	- Improves wound healing - No conclusive histological data on infected wounds anti-biofilm action [45] - inactivated by endogenous, bacterial proteases and sub-physiological salt concentrations. [45,46,47,48,49]
Kim DJ et al., 2014 [50]	SHAP1	In vivo murine model with *S. aureus* (ATCC 29213) infected wounds; topical shap1 vs. LL-37 vs. PBS	Promote and accelerate wound healing	- activation EGFR pathway - migliore riepitelizzazione rispetto a PBS e LL-37	- SHAP1 more resistant to protease and wound salt environment than LL-37 - LL-37 showed no difference in wound area compared to PBS, endogenous protease role
Chung EMC et al., 2017 [51]	DRGN-1	(1) in vivo murine model with *S. aureus* infected wound; Topical DRGN-1 vs. VK25 vs. LL-37 vs. PBS (2) in vivo murine model non-infected, Topical DRGN-1 vs. VK25 vs. PBS	(1–2) Wound healing significantly faster with DRGN-1, wound size considered	(1) skin layers were completely rehabilitated	- (1–2) DRGN-1 accelerates wound healing in both infected and non-infected wounds - (2)direct action on re-epithelialisation - (1) More effective than monotherapy with LL-37 - EGFR-STAT1/3 pathway activation - Anti-biofilm activity and antibacterial activity through membrane permeabilization
Song X. et al., 2020 [52]	DMS-PS2	In vivo murine model with MRSA infected wounds; Topical MDS-PS2 vs. untreated	DMS-PS2 improved wound healing	Not available, clinically increased rate of re-epithelialisation	- Broad-spectrum antimicrobial activities - Low toxicity mammalian blood - Important anti-biofilm action - Strong inhibition of bacterial growth
	Cell-free supernatant (CFS) of Lactobacillus plantarum USM8613	(1) porcine skin wound model infected with *S. aureus*; CFS vs. untreated (2) in vivo murine model infected with *S. aureus*; CFS vs. untreated control	(2) CFS enhanced wound contraction percentage (54%)	(2) accelerated keratinocyte migration over the wound edge towards the centre area over time (2) achieved better wound closure and complete re-epithelisation	- (1) lower bacterial count with CFS - (1) reduced biofilm thickness - (2) CFS increased the immune response (β-defensin), cytokine and chemokine production
Sojka M. et al., 2016 [53]	Def-1	In vitro Lubbock chronic wound biofilm model, *S. aureus* among other bacteria	Not available	Not available	- reduced the viability of *S. aureus* - possible role in controlling biofilm in chronic wounds
TOPICAL					
Huang J. et al., 2021 [54]	Octenidine dihydrochloride	In vivo murine model with MRSA infected skin wound	Accelerated healing and reduced bacterial counts versus control (PBS)	- Reduction of inflammatory cells - More mature collagen fibres - Well-defined epithelisation	- Useful for difficult to treat chronic wound - Possible adjuvant therapy
APDT					
Simonetti et al., 2011 [55]	RLP068/Cl	In vivo murine model with MRSA-infected wound; RLP068/Cl + aPDT (689 nm) vs. untreated vs. teicoplanin i.p.	Better results in wound healing with RLP068/CI	- RLP068/CI complete re-epithelialisation	- RLP068/CI Faster than teicoplanin in controlling infection - Better re-epithelisation than teicoplanin
Mirzahosseinipour M. et al., 2020 [56]	Curcumin encapsulated in silica nanoparticles (CEN)	In vitro human dermal fibroblast culture infected with *S. aureus*; CEN + APDT (465 nm) vs. curcumin vs. untreated	CEN Improved human fibroblast activity	the denuded region of wounds treated with curcumin and CEN was narrower than that of untreated wounds (in vitro scratch assay)	- reduction of planktonic bacteria and bacterial biofilm production - no significant fibroblast toxicity - lack of in vivo studies
Lin et al., 2020 [57]	ALA	3 patients with chronic leg ulcers resistant to conventional therapy (*S. aureus* isolated 1 patient); ALA + APDT	Clinically evident improvement without recurrences for 29 months	Not available	- the only study on patients with chronic ulcers and wound healing assessment - no bacteria isolated after treatment - lasting remission - probable direct action on wound healing, (IL-6 dependent migration of keratinocytes in vitro)
Nafee et al., 2013 [58]	Hypericin nanoparticles (HN)	In vivo murine model with MRSA infected wound; HN vs. Hypericin vs. untreated	HN showed faster wound healing	better epithelialization, keratinization, and development of collagen fibres	- direct effect on wound healing - in vitro excellent biofilm inhibition
Pérez et al., 2021 [59]	Methylene Blue (MB)-aPDT	In vivo murine model with *S. aureus* ATCC29213 infected wound; Topical MB-APDT vs. mupirocin (MU) vs. MB-APDT + MU vs. untreated	MB-aPDT improves quick mild wound contraction at 24 h, better wound healing (reduction of size, crust loss) and cosmetics results (no scar).	mild acanthosis and mild undulation of the epidermis, a thicker dermis with moderate dermal fibrosis and more dilated follicles with abundant keratin and granulomatous inflammation.	- MB-aPDT provided best clinical healing - MU enhances antimicrobial activity but not improved relevantly wound healing - No synergistic effects

CNS: coagulase-negative staphylococci, PBS: phosphate-buffered saline, EGFR: epidermal growth factor receptor, FGF-2: fibroblast growth factor, 2 DLP: defensin-like peptide, DMS-PS2: dermaseptin peptide2, ALA: aminolevulinic acid.

## Data Availability

The data presented in this study are available on request from the corresponding author.
